# Migration Study of Dielectrophoretically Manipulated Red Blood Cells in Tapered Aluminium Microelectrode Array: A Pilot Study

**DOI:** 10.3390/mi14081625

**Published:** 2023-08-17

**Authors:** Muhammad Izzuddin Abd Samad, Darven Raj Ponnuthurai, Syazwani Izrah Badrudin, Mohd Anuar Mohd Ali, Mohd Azhar Abdul Razak, Muhamad Ramdzan Buyong, Rhonira Latif

**Affiliations:** 1 Institute of Microengineering and Nanoelectronics (IMEN), Universiti Kebangsaan Malaysia (UKM), Bangi 43600, Selangor, Malaysia; p114015@siswa.ukm.edu.my (M.I.A.S.); p126284@siswa.ukm.edu.my (D.R.P.); p130001@siswa.ukm.edu.my (S.I.B.); muhdramdzan@ukm.edu.my (M.R.B.); 2 School of Electrical Engineering, Universiti Teknologi Malaysia (UTM), Skudai 81310, Johor, Malaysia; mohdanuar.mdali@utm.my (M.A.M.A.); mohdazhar@utm.my (M.A.A.R.)

**Keywords:** dielectrophoresis, tapered aluminium microelectrode array, red blood cell migration

## Abstract

Dielectrophoresis (DEP) is one of the microfluid-based techniques that can manipulate the red blood cells (RBC) for blood plasma separation, which is used in many medical screening/diagnosis applications. The tapered aluminium microelectrode array (TAMA) is fabricated for potential sensitivity enhancement of RBC manipulation in lateral and vertical directions. In this paper, the migration properties of dielectrophoretically manipulated RBC in TAMA platform are studied at different peak-to-peak voltage (Vpp) and duration supplied onto the microelectrodes. Positive DEP manipulation is conducted at 440 kHz with the RBC of 4.00 ± 0.2 µm average radius attracted to the higher electric field intensity regions, which are the microelectrodes. High percentage of RBC migration occurred at longer manipulation time and high electrode voltage. During DEP manipulation, the RBC are postulated to levitate upwards, experience the electro-orientation mechanism and form the pearl chains before migrating to the electrodes. The presence of external forces other than the dielectrophoretic force may also affect the migration response of RBC. The safe operating limit of 10 Vpp and manipulation duration of ≤50 s prevent RBC rupture while providing high migration percentage. It is crucial to define the safe working region for TAMA devices that manipulate small RBC volume (~10 µL).

## 1. Introduction

Blood plasma is the liquid portion of blood that comprises mainly water and other components like immunoglobulin, electrolytes, glucose, clotting factors, electrolytes and hormones. The blood plasma’s role in circulating biomarkers allows medical screening and diagnosis of cancer, Alzheimer’s, sepsis and infectious diseases [1,2,3]. The haemolysis of red blood cells (RBC) in blood plasma have been reported to interfere with direct biomarkers detection and have become a source of variability to the biomarker signal [4,5]. This has caused errors in laboratory medicine, accounting for up to 70% of sample rejection [6]. Therefore, it is imperative to separate RBC from blood plasma at the site of blood withdrawal in order to attain haemolysis-free sample or minimize red blood cells contamination.

Conventionally, the centrifugation approach is used to separate and extract blood plasma from large volumes of blood [7]. Although the centrifugation-based cell processing method is considerably efficient to be applied in medical research and clinical laboratories, RBC haemolysis may occur due to the exposure of blood to both air and high suction pressures [8]. Centrifugation may lead to increase in haemolysis during blood plasma separation. To circumvent this limitation, miniaturised microfluid-based approach has been proposed for blood plasma preparation [9]. The centrifugation-free system ensures a continuous flow process with low volume of blood, leading to less damaged RBC. The method provides an efficient RBC removal/separation prior to the next processing stages, subsequently reducing biomarker measurements variability and thus providing reliable detection of circulating biomarkers. Numerous microfluid-based methods have been introduced to separate RBC from whole blood that implement either micro-filtration [10,11,12], acoustic forces [13,14,15] or dielectrophoretic [16,17] technique.

Dielectrophoresis (DEP) is a manipulation technique that can be implemented to mobilise dielectric particles in a fluidic medium like RBC from one region to another. The application of non-linear electric fields onto DEP microelectrodes will exert forces onto the dielectric particles, causing them to migrate in the area between the microelectrodes [18]. Earlier design uses larger volume of RBC [19] or yeast cells [20], and the particles can be manipulated to travel to a larger distance. Recent design is usually made smaller in size and employs planar array of microelectrodes to control transportation of individual microparticles [21]. In [22], the curved microelectrode array was developed, and the dielectrophoresis method was implemented to study breast cancer cells deformation. The current design mainly caters to dielectrophoretically manipulated particles in microliter volume range [23,24].

Recently, the tapered aluminium microelectrode array (TAMA) has been designed with 70° slope to enhance the electric field distribution specifically at the two spots of the microelectrodes’ sidewall [25], which could accelerate the magnitude of particle velocities [26,27]. In the early invention, the TAMA platform was not used for DEP manipulation but only to evaluate the particles’ electrophysiological properties based on the input frequency. After half a decade, TAMA was finally applied in numerous particle manipulation studies such as for the polystyrene microbeads [26], red blood cells [27], ESKAPE bacteria [28], extracellular vesicles [29] and keratinocyte cells [30]. Highly responsive DEP manipulation without Joule heating has been achieved from TAMA. In [25], the manipulated particles can be driven laterally and vertically at the same time by which the invention could promote high selectivity and sensitivity of cell manipulation.

In this paper, the migration profile of the dielectrophoretically manipulated RBC in TAMA is presented. TAMA is designed to manipulate human blood of finger-prick volume up to a few microlitres. It is fabricated on a silicon substrate with aluminium as the microelectrodes. Human blood is diluted in deionised water, and the solution mixture is then dropped onto the fabricated TAMA. The cell radius and polarisation factor for RBC are inspected. A peak-to-peak input voltage (Vpp) at DEP operating frequency is applied onto TAMA microelectrodes, and the voltage-time behaviour of RBC manipulation is studied. The upper voltage limit and maximum manipulation time before RBC membrane started to break down and ruptured are investigated.

## 2. Theory

In 1950, Herbert Pohl observed the exerted DEP force $F_{DEP}$ on dielectric particles surrounded by a conducting liquid medium due to the application of non-linear electric fields onto two metallic electrodes [18]. The applied $F_{DEP}$ onto homogeneous spherical or non-spherical particles induces translational movement of the particles that reside in between two electrodes. $F_{DEP}$ is defined as

(1) $$F_{DEP}=2\pi \varepsilon_{m}r^{3}Re\left[f_{CM}\left(\omega \right)\right]\;\nabla \left|E_{rms}^{2}\right|$$

where $\varepsilon_{m}$ is the permittivity of the suspending medium surrounding the dielectric particles, r represents the particle’s radius, $Re\left[f_{CM}\left(\omega \right)\right]$ is the real part of the Clausius–Mossotti factor (CMF), $E_{rms}$ is the root-mean-square value of the applied electric field to the electrodes and ω is the angular frequency (ω=2πf), where f is the frequency of the applied electric field [31,32]. The $Re\left[f_{CM}\left(\omega \right)\right]$ CMF polarisation factor values are used to predict the pathway of the manipulated cell particles, which is based on the interaction between the particles’ and medium’s polarisation [25,26]. If $Re\left[f_{CM}\left(\omega \right)\right]$ is positive, the manipulated cell particles would get attracted to a high-intensity electric field region and would be denoted as positive DEP response (pDEP). Meanwhile, the manipulated cell particles repel the high-intensity electric field region and driven towards low electric field density when $Re\left[f_{CM}\left(\omega \right)\right]$ is negative, implying negative DEP manipulation (nDEP). The manipulated cells remain static when $Re\left[f_{CM}\left(\omega \right)\right]$ is zero, indicating equal polarization of the manipulated cells and medium. The applied frequency at this point is known as the crossover frequency, whereby there are almost no forces acting on cells.

In this study, a simplified single-shell model is employed to estimate the CMF polarisation factor $f_{CM}\left(\omega \right)$ of RBC. In Equation (2), CMF is a function of the frequency of the electric field, and it is defined by the complex permittivity of the particle $\varepsilon_{p}^{*}$ and the medium $\varepsilon_{m}^{*}$ [33].

(2) $$f_{CM}\left(\omega \right)=\frac{\varepsilon_{p}^{*}-\varepsilon_{m}^{*}}{\varepsilon_{p}^{*}+2\varepsilon_{m}^{*}}$$

The complex permittivity can be written as in Equation (3) with σ represents the electric conductivity and i is the imaginary unit $\sqrt{-1}$.

(3) $$\varepsilon^{*}=\varepsilon -i\frac{\sigma }{\omega }$$

Biological cells like RBC are not naturally homogeneous spheres, and there is a high contrast in conductivity between the cytoplasm and membrane of the red blood cell. Therefore, a core–shell model is considered, and a mixing equation is used to attain the effective complex permittivity of cell particles, $\varepsilon_{p,eff}^{*},$ in Equation (4).

(4) $$\varepsilon_{p,eff}^{*}=\varepsilon_{mem}^{*}\frac{{\left(\frac{r}{r_{o}}\right)}^{3}+2\left(\frac{\varepsilon_{cyt}^{*}-\varepsilon_{mem}^{*}}{\varepsilon_{cyt}^{*}+2\varepsilon_{mem}^{*}}\right)}{{\left(\frac{r}{r_{o}}\right)}^{3}-\left(\frac{\varepsilon_{cyt}^{*}-\varepsilon_{mem}^{*}}{\varepsilon_{cyt}^{*}+2\varepsilon_{mem}^{*}}\right)}$$

The $\varepsilon_{cyt}^{*}$ and $\varepsilon_{mem}^{*}$ are the complex permittivities of the cytoplasm (core) and membrane (shell), respectively. The particle’s radius, r, considers the radius of the entire cell (shell+core) while $r_{o}$ is the radius of the cytoplasm (core) [7,33].

## 3. Materials and Methods

### 3.1. Fabrication of TAMA

**Figure 1:**
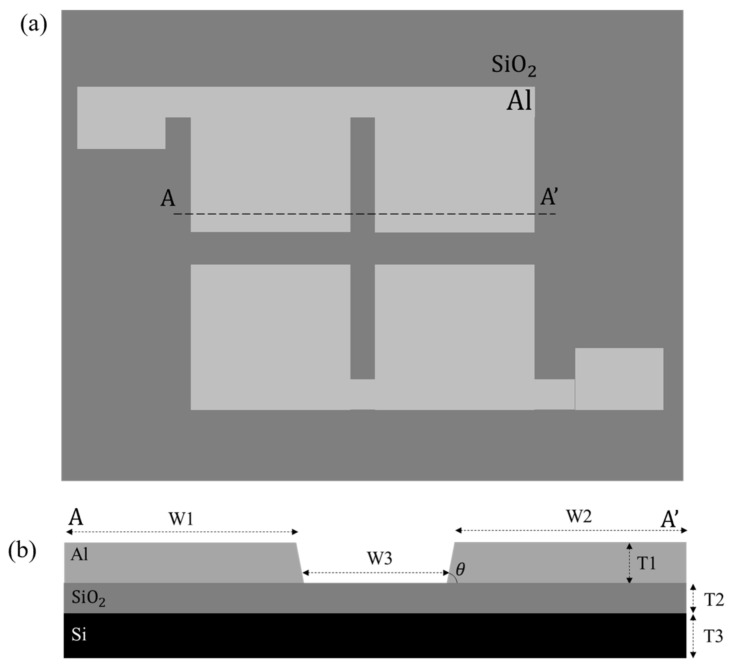
Schematic diagram of TAMA from (**a**) top and (**b**) cross-section at AA’.

The top view and cross-sectional view of TAMA at AA’ are depicted in Figure 1. The corresponding geometrical dimensions for TAMA are tabulated in Table 1. The interelectrode gap width (W3) of 80 µm is the spacing distance between the microelectrodes. θ describes the slope angle of the microelectrode edge. The fabrication of tapered aluminium microelectrode array starts with the silicon substrate. First, the silicon dioxide layer (SiO_2_) of 1 µm thickness is deposited on silicon substrate using the plasma-enhanced chemical vapour deposition (PECVD) process. SiO_2_ serves as the electrical insulation layer. Next, the titanium/titanium nitride (Ti/TiN) seed layer of 60 nm/30 nm thickness is deposited on SiO_2_ as an adhesion layer for the metal microelectrode. After that, 4 μm thickness of aluminium (Al) layer is deposited and patterned to be the metal microelectrodes for TAMA. In the fabrication process, both Ti/TiN seed layer and the metallic Al electrode layer are deposited via physical vapor deposition (PVD) method, and the layers are patterned photolithographically to define the square array pattern for the microelectrodes. Finally, the Ti/TiN and Al layers are etched in reactive ion etching-inductively coupled plasma (RIE-ICP) etcher along with the advanced plasma resist strip technique to create the metal edge etching. A previous paper can be referred to for further information on the fabrication of 70° microelectrode edge slope [31]. The schematic diagram in Figure 1 only shows two pairs of microelectrodes for simplification. There are 5–6 microelectrode pairs in our fabricated TAMA. The dielectrophoretic response at the region of interest are measured independent of each other.

### 3.2. Sample Preparation

**Figure 2:**
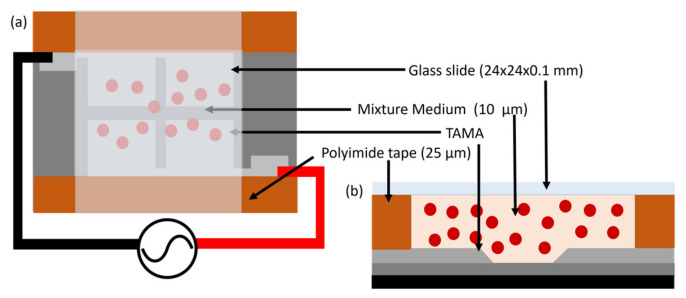
The schematic view of the prepared solution chamber on top of TAMA platform: (**a**) top view and (**b**) side view.

Approximately 1 µL of fresh blood is diluted in 99 µL deionised water, and 10 µL of the solution mixture is dropped on top of the TAMA platform. Then, the microelectrodes are covered with a 24 mm × 24 mm × 0.1 mm cover slide. Polyimide tape of 20 µm thickness is taped on the sides of the TAMA platform to create a fluidic chamber. The schematic illustrations in Figure 2a,b demonstrate the top view and the cross-sectional view of TAMA with the prepared solution mixture and the cover slide chamber. The small volume used for the solution mixture and the cover slide help to prevent the solution from escaping the fluidic chamber.

### 3.3. Physiological Inspection of RBC

A physical inspection has been conducted to measure the red blood cells’ diameter. It is one of the critical parameters to be used in identifying the CMF polarisation factor for RBC. The prepared sample has been placed under 50× magnification of optical microscope lens (Olympus STM6, Tokyo, Japan) to observe and measure the RBC’s diameter. The optical image of cell is then captured by Dino-lite Digital Microscopy software via Dino-Lite Eyepiece Camera. The captured image and video are processed by DinoCapture 2.0 software where the physical parameters of RBC are measured automatically.

The average radius of RBC and its cytoplasm are then estimated and used in the single shell model to simulate the CMF polarisation factor for RBC. In MyDEP simulation tool, the dielectric properties of the suspending fluid medium (blood plasma) and RBC have been considered with the frequency f of the applied electric field from 100 kHz to 10 MHz. The simulation will predict the crossover frequency and the movement pattern of RBC during DEP manipulation. The dielectric properties of the medium suspension and RBC are listed in Table 2. Indirectly, the physical inspection also identifies the viability of RBC; either the RBC is still healthy or experiencing lysis during sample preparation. If the RBC is lysed, it is considered as a dead cell.

### 3.4. RBC Dielectrophoresis Measurement and Quantification

**Figure 3:**
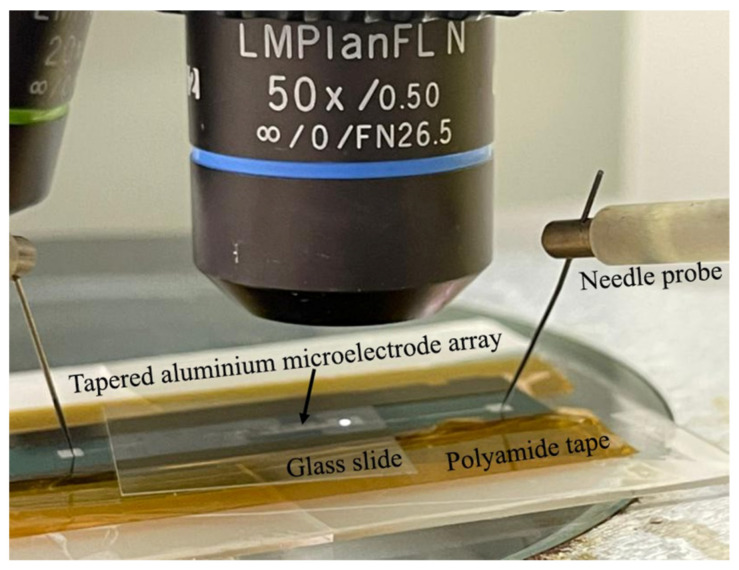
The measurement setup for DEP manipulation of RBC in TAMA.

The function generator is used to supply the alternating peak-to-peak voltage to the microelectrodes of TAMA via the needle probers (Figure 3). At the chosen DEP operating frequency, the manipulation of RBC in TAMA is observed and recorded for 60 s at a constant input voltage of 5 Vpp. Afterwards, the input voltage is increased by 1 Vpp until it reaches 10 Vpp. In each case, we follow the dielectrophoretic movement of RBC, and every 10 s time frame was acquired and evaluated. The Dino-Lite Eyepiece Camera is mounted on the microscope eyepiece to record the movement of RBC during DEP manipulation process.

**Figure 4:**
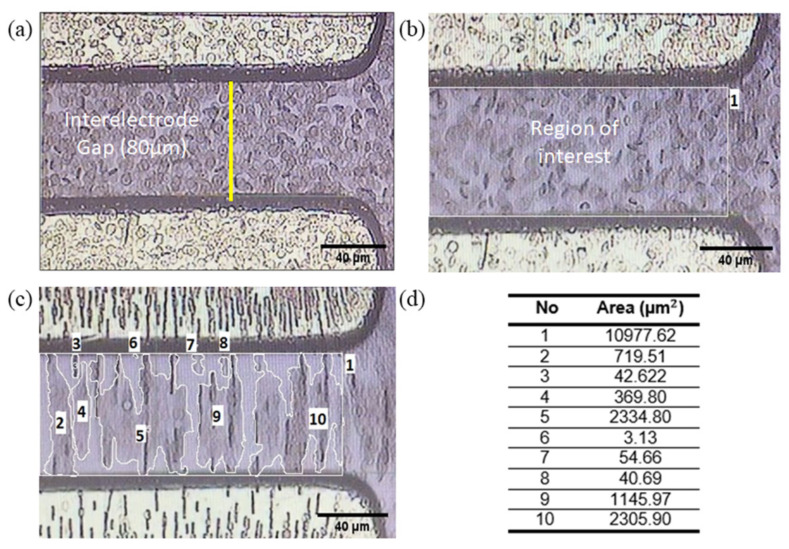
The area quantification method to estimate RBC migration: (**a**) The calibrated raw image setting of the interelectrode region from TAMA; (**b**) The region of interest (ROI) area for DEP manipulation, $a_{0}$; (**c**) The measured suspended areas of RBC islands in ROI; and (**d**) The corresponding labelled list of the measured areas. Areas labelled 2–10 total up to be $a_{t}$, while the area number 1 is $a_{0}$.

The still image sequences in the recorded video of RBC movement at certain DEP manipulation times have been examined and analysed. Several different methods could be applied to quantify RBC migration such as the grid, area [32] or pixel quantification method. In our work, we calculate the percentage of RBC migration ($n_{RBC}$) based on the distribution area of red blood cells at certain time frames. Firstly, the raw image needs to be calibrated from pixel scale into micrometre scale range (Figure 4a). Then, the 300 × 240 pixels of the region of interest (ROI) that is located at the interelectrode gap of TAMA is chosen as the reference area ($a_{0}$) for RBC manipulation (Figure 4b). After that, the red blood cells distribution area ($a_{t}$) at a certain manipulation time is estimated manually via a freehand drawing tool (Figure 4c). The area of each RBC island labelled from number 2 to 10 are summed up to be $a_{t}$ (Figure 4d). Finally, the percentage of RBC migration $n_{RBC}$ at different DEP conditions is calculated using Equation (5). The quantification of RBC migration in static mode is quite challenging, but it may provide some valuable insights of the migration process. The average number of trials for this experiment is five in each case, and the goal is to increase the migration of RBC from the region of interest with the cells retaining their healthy condition.

(5) $$n_{RBC}=\frac{a_{0}-a_{t}}{a_{0}}\times 100\%$$

## 4. Results and Discussions

### 4.1. Electrophysiological Properties of RBC

**Figure 5:**
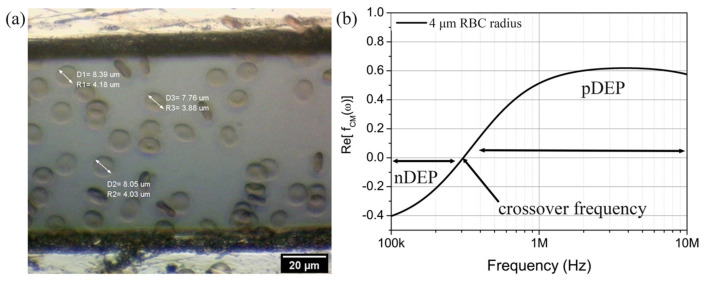
(**a**) The electrophysiology of RBC under the optical microscope of 50× magnification lens. (**b**) The CMF polarisation factor plot of RBC on semilogarithmic frequency (100 kHz to 10 MHz) for an average radius of RBC, r = 4.00 ± 0.2 µm.

The physical inspection of RBC under 50× magnification of the optical microscope lens is shown in Figure 5a. The structure of RBC is identified to be in the form of a biconcave disk. The average radius of cells is measured to be r = 4.00 ± 0.2 µm. The thickness of RBC plasma membrane is approximately 0.01 µm giving cytoplasmic radius of ~$r_{o}$ = 3.99 µm. Based on the measurement of radius and diameter, the cells are classified as healthy specimens and within the standard range of normal RBC [33].

In Figure 5b, the CMF polarisation factor of RBC was simulated using MyDEP software, which predicts the dipole moment of RBC that is either repelled or attracted towards the high-intensity electric field regions from frequency f of 100 kHz to 10 MHz. The properties of blood plasma medium and RBC in Table 2 have been used in the single shell model. The real part of the CMF polarisation factor, $Re\left[f_{CM}\left(\omega \right)\right],$ ranges from −0.4 to 0.6, and the crossover frequency has been found to be at ~307 kHz. Negative DEP effect, nDEP, on RBC occurs at 100 kHz–307 kHz as indicated by $Re\left[f_{CM}\left(\omega \right)\right]<0$. It is expected for the RBC to experience negative dielectrophoretic forces in the microelectrode array, whereby the cells are repelled towards the weakest electric filed regions. In contrast, $Re\left[f_{CM}\left(\omega \right)\right]>0$ in the 307 kHz–10 MHz frequency range, suggesting positive DEP (pDEP) effect. In this frequency range, the RBC movement pattern would be towards the strongest electric field regions in TAMA. In this work, we set the operating frequency for DEP manipulation to be at $Re\left[f_{CM}\left(\omega \right)\right]=0.2$, that is, equivalent to 440 kHz. We would like to induce positive dielectrophoretic forces on RBC in the next stages of the experiments.

### 4.2. pDEP Manipulation of RBC

**Figure 6:**
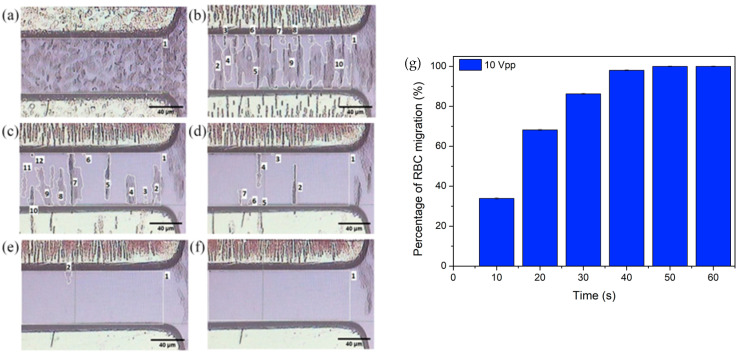
Positive DEP manipulation of RBC at 10 Vpp of voltage applied onto the TAMA microelectrodes with different manipulation duration: (**a**) 0 s, (**b**) 10 s, (**c**) 20 s, (**d**) 30 s, (**e**) 40 s and (**f**) 50 s. (**g**) The calculated percentage of RBC migration against manipulation time, from 0 s–60 s at constant applied voltage of 10 Vpp. Supplementary Material Video S1.

An electrical signal of 10 Vpp and 440 kHz is applied on the microelectrodes of TAMA for 60 s, and the migration of RBC from the region of interest is quantified. Figure 6 demonstrates the positive DEP movement of RBC during 0 s–60 s of manipulation time. Before the application of the electrical signal, the RBCs are distributed in a fairly uniform manner at the region of interest, i.e., within the interelectrode spacing (Figure 6a). Upon the application of voltage, the RBCs move towards the microelectrodes that possess higher electric field intensity compared to the region of interest area. In Figure 6b–d, it is observed that the RBC have created suspended distribution islands in the region of interest before migrating. The RBC have been observed to be fully migrated at 50 s of manipulation time (Figure 6f).

The percentage of RBC migration $n_{RBC}$ at different manipulation time frames have been calculated from the measured areas of RBC distribution islands and histogram plotted in Figure 6g. Rapid RBC migration of 33.7 ± 0.3% is observed within the first 10 s. More RBCs are migrating to the microelectrodes with longer application of electric field duration. 100% of RBCs in ROI have been successfully manipulated by the positive DEP forces to migrate to the electrodes by 50 s. The voltage–time performance analysis is essential in our research in order to elucidate the responsiveness of TAMA platform in driving the RBC off from the interelectrode gap. Through the optical observation, TAMA is capable of provoking strong DEP manipulation at 10 Vpp and 440 kHz electrical signals.

**Figure 7:**
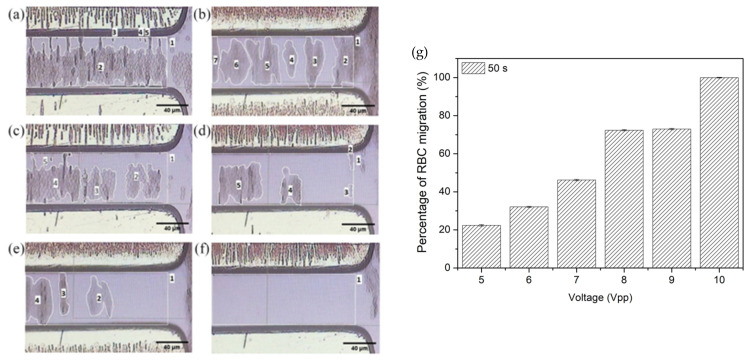
Positive DEP manipulation of RBC at 50 s of manipulation duration with different voltage applied onto the TAMA microelectrodes: (**a**) 5 Vpp, (**b**) 6 Vpp, (**c**) 7 Vpp, (**d**) 8 Vpp, (**e**) 9 Vpp and (**f**) 10 Vpp. (**g**) The calculated percentage of RBC migration against the applied voltage at a constant 50 s of manipulation time.

Figure 7 demonstrates the RBC migration performance at different input voltages varying from 5 Vpp to 10 Vpp with 1 Vpp increment. The manipulation time of 50 s has been chosen even though smaller manipulation duration could be used to avoid air bubbling, Joule heating and cell disintegration from occurring. In our measurement, the disintegration of RBC and air bubbling during DEP manipulation at the chosen condition were undetected. As the voltage increases from 5 Vpp to 9 Vpp (Figure 7a–e), more RBC migration has been observed for higher applied voltage to the microelectrodes with a decrease in distribution areas of the suspended RBC islands. At 10 Vpp in Figure 7f, all RBCs have fully migrated to the electrodes.

Figure 7g shows the histogram plot of the percentage RBC migration $n_{RBC}$ with respect to different electrode voltages at a constant manipulation time of 50 s. The smallest migration percentage has been recorded at 5 Vpp with 22.4 ± 0.4% while a total of 100% RBC migration is achieved at 10 Vpp. Higher voltage may provide higher electric field strength, and thus, the RBC may experience stronger DEP manipulation force. Consequently, higher number of RBC are induced to migrate faster at higher voltage.

Overall, we found that the increase of both magnitude and duration of the applied electrode voltage can significantly increase the migration performance of RBC in TAMA platform. At the region of interest, the migrated RBC have left the interelectrode spacing area, leaving behind the blood plasma and other components. This separation would take out RBC from whole blood, and in a continues flow process, the remaining blood components at the interelectrode spacing can be collected for blood plasma preparation. The 100% migration of RBC would prevent RBC contamination during blood plasma collection. In the experiment, we have also found that the RBC start to disintegrate at 10 Vpp and manipulation time of more than 60 s. Many of the RBC were ruptured or experienced lysis probably due to the higher magnitude and longer DEP force acting on the cells. At this point, it is crucial to set the limit of amplitude and duration of the applied electrical voltage onto the microelectrodes and simultaneously attempt to achieve high and rapid migration of RBC. There should be a compromise for achieving fast, 100% RBC migration and minimising RBC haemolysis at the same time. In our work, for finger-prick blood volume and 80 µm of interelectrode TAMA spacing, the maximum voltage that could be applied is 10 Vpp with time duration of less or equal to 50 s, to safely manipulate RBC with high migration percentage and low RBC damage.

### 4.3. The Electrokinetic Mechanism

**Figure 8:**
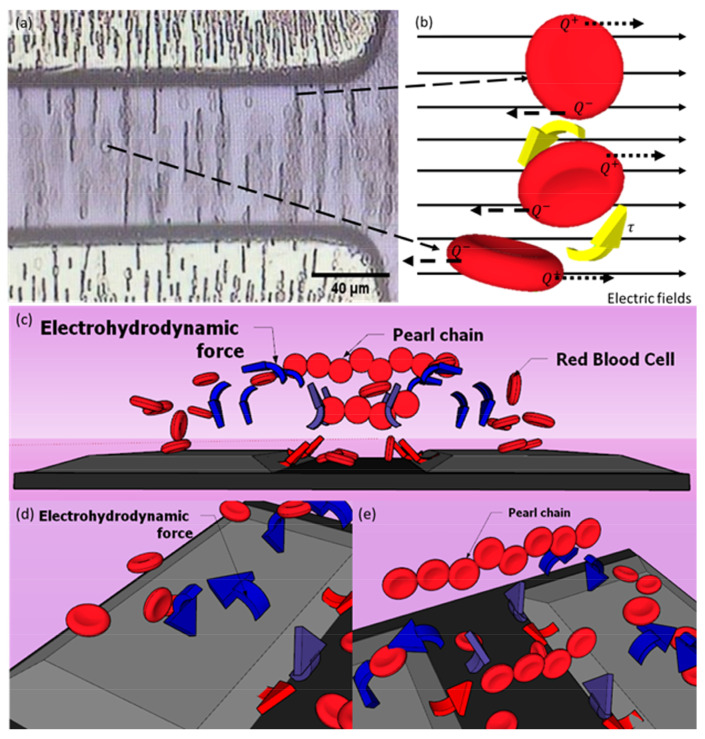
The schematic illustration of the electrokinetic phenomenon occurring in DEP manipulation of RBC in TAMA platform at 10 Vpp and 10 s of manipulation time. (**a**) Strings of pearl chains formation by RBC. (**b**) The RBC electro-orientation mechanism by which the cells are experiencing torque forces due to the central charges accumulating towards high and low electric field potentials. (**c**) The manipulated RBC being levitated upward and forming pearl chains at the same time. (**d**) The up-thrust electrohydrodynamic forces acting on RBC. (**e**) The pearl chain formation of the red blood cells due to their dipole–dipole interaction.

During DEP manipulation, we observed (1) the formation of pearl chains by the red blood cells as shown in Figure 8a. We have also witnessed electro-orientation where the polarised RBC (2) orient themselves along the direction of the applied electric field as depicted in Figure 8b. Additionally, these cells seem to be (3) levitated upwards in the suspended medium before migrating to the TAMA microelectrodes (Figure 8c). These three different observed phenomena can be explained by the presence of dipole–dipole interaction between the RBC cells, dipole–electric field interaction and electrohydrodynamic (EDH) forces in the medium suspension.

The electro-orientation of RBC happens due to the non-spherical structure of the cells. During particle polarisation, the cells’ dipole moment would try to align themselves along the non-uniform electric field [18]. Theoretically, the cells’ charges accumulate along the major axis of the non-spherical particles. Torque forces (τ) are induced and changed according to the accumulation of positive (Q^+^) and negative (Q^−^) charges within RBC with respect to the high and low potentials of the electric field as illustrated in Figure 8b. This mechanism could be advantageous in rearranging particles according to the desired pattern for specific applications. For example, Zhang et al. have attempted this DEP alignment technique to polarise and rearrange the piezoelectric zinc oxide microparticles on a polydimethylsiloxane elastomer substrate [34].

Two electrokinetic mechanisms are illustrated in Figure 8c whereby the manipulated red blood cells in the suspended medium are shown to levitate upwards and migrate to the top of TAMA electrodes while some of the cells form pearl chains. The particle levitation usually occurs due to the exerted electrohydrodynamic (EDH) forces in the medium suspension, induced by the electrothermal vortex [35,36] and buoyancy forces [37]. In Figure 8d, the warmer medium suspension region, which is close to the microelectrode edge slope surface (red arrow), is replaced by the cool medium (blue arrow) to achieve a state of thermal equilibrium (violet arrow) in the medium suspension. The liquid circulation is a result of the electrothermal vortex, building up the up-thrust EDH forces acting on RBC.

Figure 8e illustrates the red blood cells forming the pearl chains during DEP manipulation. The RBC experience dipole–dipole interaction between the cells, where the dipole of a cell combines with the dipole of a neighbouring cell, resulting in a long cell string [38,39]. The pearl chain formation could also interrupt the electro-orientation mechanism of the cells by forcing the dipole particles to align end to end in the same direction [18]. The resultant of dipole–dipole cell motion depends on the electric field gradient and the type of dipole. In our work, we have observed the formation of pearl chains by RBC exactly after the application of input voltage, and the long-string RBC pearl chains were extended as the manipulation time increased, before migrating to the TAMA microelectrodes. The RBC located close to the microelectrodes would quickly move towards the microelectrodes. The ones at the middle of the interelectrode spacing would usually line up forming clusters of pearl chains and reorientating themselves before eventually all migrated towards the microelectrode. No isolated case of RBC is observed whereby almost all of them were moving to the microelectrodes or taking part in forming the pearl chain lines in the middle of ROI. Once the voltage is off, the pearl chains disassemble.

## 5. Conclusions

TAMA microelectrodes have been fabricated, and the voltage–time response of RBC in DEP manipulation has been studied. A positive DEP pattern has been measured from RBC at DEP operating frequency of 440 kHz with the cells in the suspended medium moving towards the electrodes. The higher percentage of RBC manipulation occurred at higher voltage supply and longer manipulation duration. The levitation of RBC during DEP manipulation suggests the presence of electrohydrodynamic (EDH) forces in the medium suspension other than the dielectrophoretic forces. The electro-orientation mechanism demonstrated by RBC insinuates the existence of interaction between the cells’ dipole moment and the applied electric field. Additionally, the formation of pearl chains by RBC cells implies that there are dipole–dipole interactions between the RBC cells. We postulate that the presence of EDH forces and cell interactions may aid the dielectrophoretic forces in manipulating the red blood cells. The balanced forces within the chamber should be examined in greater detail to quantify the observed levitation and investigate the influence of temperature. Haemolysis-free blood plasma could be potentially attained by efficiently and rapidly separating RBC from the whole blood and optimising the application of voltage magnitude and manipulation duration to prevent RBC lysis. The absence of RBC and RBC lysis would improve the blood plasma’s function of circulating biomarkers for more accurate medical screening and diagnosis. The future work will focus on the microfluidic approach in blood plasma components collected at the interelectrode region.

## Figures and Tables

**Table 1:** The geometrical dimensions of TAMA.

Parameter	Dimension
Microelectrode width, W1	1000 μm
Microelectrode width, W2	1000 μm
Interelectrode spacing, W3	80 μm
Microelectrode edge slope, θ	70°
Microelectrode thickness (Al), T1	4 μm
Insulation layer thickness (SiO_2_), T2	1 μm
Substrate thickness, T3	600 μm

**Table 2:** The properties of blood plasma medium and healthy living RBC.

Category	Parameter	Value	Reference
Medium suspension	Relative permittivity for $\varepsilon_{m}$	80	[7]
	Conductivity, $\sigma_{m}$	45 mS/m	[7]
Living RBC	Total cell radius, r	4.00 µm	Own data
	Cytoplasma radius, $r_{o}$	3.99 µm	Own data
	Cytoplasma conductivity, $\sigma_{cyt}$	310 mS/m	[7]
	Relative permittivity for $\varepsilon_{cyt}$	59	[7]
	Shell electrical conductivity, $\sigma_{mem}$	1 µS/m	[7]
	Shell relative permittivity for $\varepsilon_{mem}$	4.4	[7]

## Data Availability

Please contact the corresponding author.

## Supplementary Materials

The following supporting information can be downloaded at https://www.mdpi.com/article/10.3390/mi14081625/s1, Video S1: DEP manipulation of RBC at 10 Vpp of voltage applied onto the TAMA microelectrodes with different manipulation duration.
